# Association Between End-Tidal Carbon Dioxide Pressure and Cardiac Output During Fluid Expansion in Operative Patients Depend on the Change of Oxygen Extraction

**DOI:** 10.1097/MD.0000000000003287

**Published:** 2016-04-08

**Authors:** Pierre-Grégoire Guinot, Mathieu Guilbart, Abdel Hakim Hchikat, Marie Trujillo, Pierre Huette, Stéphane Bar, Kahina Kirat, Eugénie Bernard, Hervé Dupont, Emmanuel Lorne

**Affiliations:** From the Anesthesiology and Critical Care Department (P-GG, MG, AHH, MT, PH, SB, KK, EB, HD, EL), Amiens University Hospital, Amiens, France and INSERM U1088 (P-GG, HD, EL), Jules Verne University of Picardy, Amiens, France.

## Abstract

In a model of hemorrhagic shock, end-tidal carbon dioxide tension (EtCO_2_) has been shown to reflect the dependence of oxygen delivery (DO_2_) and oxygen consumption (VO_2_) at the onset of shock. The objectives of the present study were to determine whether variations in EtCO_2_ during volume expansion (VE) are correlated with changes in oxygen extraction (O_2_ER) and whether EtCO_2_ has predictive value in this respect.

All patients undergoing cardiac surgery admitted to intensive care unit in whom the physician decided to perform VE were included. EtCO_2_, cardiac output (CO), blood gas levels, and mean arterial pressure (MAP) were measured before and after VE with 500 mL of lactated Ringer solution. DO_2_, VO_2_, and O_2_ER were calculated from the central arterial and venous blood gas parameters. EtCO_2_ responders were defined as patients with more than a 4% increase in EtCO_2_ after VE. A receiver-operating characteristic curve was established for EtCO_2_, with a view to predicting a variation of more than 10% in O_2_ER.

Twenty-two (43%) of the 51 included patients were EtCO_2_ responders. In EtCO_2_ nonresponders, VE increased MAP and CO. In EtCO_2_ responders, VE increased MAP, CO, EtCO_2_, and decreased O_2_ER. Changes in EtCO_2_ were correlated with changes in CO and O_2_ER during VE (*P* < 0.05). The variation of EtCO_2_ during VE predicted a decrease of over 10% in O_2_ER (area under the curve [95% confidence interval]: 0.88 [0.77–0.96]; *P* < 0.0001).

During VE, an increase in EtCO_2_ did not systematically reflect an increase in CO. Only patients with a high O_2_ER (i.e., low ScvO_2_ values) display an increase in EtCO_2_. EtCO_2_ changes during fluid challenge predict changes in O_2_ER.

## INTRODUCTION

The objective of volume-based hemodynamic resuscitation is to raise cardiac output (CO) and increase or restore the delivery of oxygen (DO_2_) required to meet the demands of oxygen consumption (VO_2_).^1^ If DO_2_ drops below a critical threshold, oxygen extraction (O_2_ER) cannot increase in proportion to demand, and VO_2_ becomes dependent on DO_2_. Before critical O_2_ER values arise, DO_2_ can decrease independently of VO_2_ (because DO_2_ exceeds VO_2_) and O_2_ER will increase with demand (as demonstrated by a progressive fall in central venous saturation [ScvO_2_]).^2^ When O_2_ER cannot rise any further, VO_2_ decreases and the body's metabolism becomes partially anaerobic (with a resulting increase in blood lactate levels).^3^ In many pathological situations, VO_2_ remains constant over a wide range of DO_2_ values as a result of adjustments in tissue oxygen uptake.^4^ ScvO_2_ is a clinically meaningful measure of tissue oxygenation,^5^ since it assesses the adequacy of DO_2_ with regard to VO_2_.^6^ Several studies have shown that ScvO_2_-based hemodynamic resuscitation is associated with lower morbidity and mortality rates during anesthesia and intensive care.^7,8^ Exhaled CO_2_ (end-tidal carbon dioxide tension, EtCO_2_) is also monitored in patients in the intensive care unit (ICU) or during anesthesia.^9^ Over short periods (and assuming a constant metabolic state), there is a qualitative relationship between EtCO_2_ and CO.^10,11^ Thus, EtCO_2_ can be used as a noninvasive, continuous measure of CO during several clinical situations with low-flow states.^10–13^ These results have not been confirmed in patients scheduled for surgery, in whom CO increased upon volume expansion (VE).^14^ One possible explanation is that patients scheduled for surgery and patients in the ICU differ in terms of systemic oxygen supply dependency. Most of the literature studies were performed in low-flow states, in which patients have been on a dependence phase between DO_2_ and VO_2_. This is probably not the case for most patients in the operating theatre. Based on a model of hemorrhagic shock in dogs, Guzman et al^12^ demonstrated that EtCO_2_ can reflect the dependence of DO_2_ and VO_2_ at the onset of shock and during hemodynamic resuscitation. Thereafter, Dubin et al^13^ confirmed the relationship between EtCO_2_, DO_2_, and VO_2_. Lastly, EtCO_2_ may be a noninvasive indicator of O_2_ER (and its surrogate ScVO_2_). Hence, the primary objective of the present study was to confirm that variations in EtCO_2_ during VE are correlated with changes in O_2_ER. We also evaluated the ability of variations in EtCO_2_ to predict a decrease in O_2_ER during VE.

## METHODS

### Ethics

The study's objectives and procedures were approved by the local independent ethics committee. Ethical approval for this study (Ethical Committee No. RNI2014-15) was provided by the Comité de Protection des Personnes Nord-Ouest II CHU—Place V. Pauchet, 80054 AMIENS Cedex 1 (Chairperson Bourgueil Thierry) on June 26, 2014. All patients received written information on the study and gave their verbal consent to participation prior to surgery. The present manuscript was drafted in compliance with the STROBE checklist for cohort studies.^15^

### Patients

This prospective, observational study started on June 30, 2014 at Amiens University Hospital's cardio vascular and thoracic ICU over a 6-month period. The inclusion criteria were any major patient over 18 years, ventilated with controlled positive ventilation, for whom the physician decided to do a VE within hours of admission to the ICU. The indications for VE were arterial hypotension (systolic arterial pressure [SAP] lower than 90 mm Hg and/or mean arterial pressure [MAP] lower than 65 mm Hg), oliguria (urine output lower than 0.5 mL/kg per h over 1 h), clinical signs of hypoperfusion (skin mottling, capillary refill time over 2 s), and arterial hyperlactatemia (arterial lactate over 2 mmol/L). The noninclusion criteria were permanent arrhythmia, chronic obstructive pulmonary disease, and acute lung injury. The exclusion criteria were spontaneous ventilation, poor echogenicity, and arrhythmia.

### Hemodynamic Parameters

An internal jugular vein central venous catheter and an arterial catheter were placed in all patients. Central venous pressure (CVP) and blood pressure were measured with a transducer zeroed at the mid-axillary line. Transthoracic echocardiography (Cx50, Philips Medical System, Suresnes, France) was performed by a physician who was blinded to the study outcomes. The left ventricular ejection fraction was measured using Simpson biplane method with a 4-chamber view. The diameter of the left ventricular outflow tract (LVOT) was measured on a long-axis parasternal view upon patient inclusion. Aortic area (SAo, in cm^2^) was calculated as π × LVOT^2^/4. The aortic velocity-time integral (VTIAo) was measured with pulsed Doppler and a 5-chamber apical view. Stroke volume (SV) (mL) was calculated as VTIAo × SAo. CO (in L/min) was calculated as SV × heart rate (HR). Mean echocardiographic parameters were calculated from 5 measurements (regardless of the respiratory cycle) and analyzed retrospectively. The intra and inter reproducibility of VTIAo measurements was tested prior to the study. Reproducibility values were 4.4 ± 3.9% and 4.4 ± 3.2%, respectively.

### Oxygenation Parameters and EtCO_2_

We recorded the ventilator settings (tidal volume, plateau pressure, and end-expiratory pressure) at baseline. Exhaled CO_2_ was continuously measured at the tip of the endotracheal tube using a CO_2_ cuvette with an integrated sensor (Drager, Luebeck, Germany). All parameters were measured on arterial and central venous blood gases. Arterial and venous blood gas levels, the lactate level, the blood hemoglobin concentration, and oxyhemoglobin saturation were assayed using an automated analyzer (ABL800 FLEX, Radiometer, Bronshoj, Denmark). Arterial oxygen content (CaO_2_) and venous oxygen content (CvO_2_) were calculated as follows: CaO_2_ = 1.34 × Hb × SaO_2_ + 0.003 × PaO_2_; CvO_2_ = 1.34 × Hb × ScvO_2_ + 0.003 × PvO_2_, where Hb is the hemoglobin concentration (in g/dL), PaO_2_ is the arterial oxygen pressure (in mm Hg), SaO_2_ is the arterial oxygen saturation (in %), PvO_2_ is the venous oxygen pressure (mm Hg), ScvO_2_ is the central venous oxygen saturation (in %), and 0.003 the solubility coefficient of oxygen. PCO_2_ gap was calculated as follow: PCO_2_ gap = PcvCO_2_ − PaCO_2_ (mm Hg).

DO_2_ and VO_2_ were calculated from arterial and central venous blood gases as follows: DO_2_ (mL/min per kg) = (CaO_2_ × 10 × CO)/weight; VO_2_ (mL/min per kg) = the arteriovenous difference in oxygen content ([C(a − v)O_2_] × CO × 10)/weight. O_2_ER was defined as VO_2_/DO_2_ ratio. Arterial and venous CO_2_ contents (CaCO_2_, CvCO_2_) were calculated according to Douglas Formula.^16^ The alveolar dead space (Vd/Vt) was estimated from EtCO_2_ and PaCO_2_ as (PaCO_2_ − EtCO_2_)/PaCO_2_.^17^

### Study Procedures

The following clinical parameters were recorded: age, gender, weight, and main diagnosis. First, a passive leg-raising (PLR) test was performed in order to evaluate the effects on SV, and assess preload status. After an equilibration period, baseline measurements of HR, SAP, MAP, diastolic arterial pressure (DAP), CVP, SV, CO, EtCO_2_, and arterial/venous blood gas levels were obtained. In the present study, VE always consisted in infusing 500 mL of lactated Ringer solution over 10 min; 10 min after VE, a second set of measurements (SAP, MAP, DAP, HR, CVP, SV, CO, EtCO_2_, and arterial/venous blood gas levels) was recorded. All patients had been sedated via the continuous infusion of propofol and were fully accustomed to mechanical ventilation. All patients underwent mechanical ventilation in volume-controlled mode with a tidal volume set to 7 to 9 mL/kg of ideal body weight, and a positive end-expiratory pressure of 5 to 8 cm H_2_O. Ventilator settings (oxygen inspired fraction, tidal volume, respiratory rate, and end positive pressure) and norepinephrine dosage were not modified during the study period.

### Statistical Analysis

We calculated that a sample of 50 patients would be sufficient to demonstrate a correlation of over 0.7 between EtCO_2_, ERO_2_, VO_2_, DO_2_, and CO. Fifty-five patients were therefore recruited, taking into account the exclusion criteria. The variables’ distribution was assessed using a Kolmogorov–Smirnov test. Data are expressed as the proportion (in %), the mean (standard deviation, SD) or the median (interquartile range), as appropriate. We measured the magnitude of EtCO_2_ variations during VE by calculating the effect size (the mean divided by the SD).^18,19^ The effect size was 0.74. Then, we calculated the coefficient of variation (CV), precision and least significant change (LSC) for EtCO_2_. LSC is the least amount of EtCO_2_ change that can be considered statistically significant; that is, the minimum percentage change between successive measurements that can be considered not due to random error and therefore representing a real change in ETCO_2_. The EtCO_2_ CV and LSC were determined in all studied patients at baseline during stable respiratory and hemodynamic conditions. The CV (95% confidence interval [CI]) was 1.8% (0.9–2.7) and the LSC (95% CI) was 2.5% (1.3–3.8). EtCO_2_ responder was defined as an increase of EtCO_2_ of more than 4% in EtCO_2_ after VE. EtCO_2_ nonresponder was defined as an increase of EtCO_2_ of <4% in EtCO_2_ after VE. This cut off correspond to LSC with its 95% CI.^20^ Fluid responder was defined as an increase of more than 15% in the SV during VE.^21^ Fluid nonresponder was defined as an increase of <15% in the SV during VE. The nonparametric Wilcoxon rank sum test, Student paired *t* test, Student *t* test, and the Mann–Whitney test were used to assess statistical significance, as appropriate. Linear correlations were tested using Pearson or Spearman rank method. A receiver-operating characteristic curve was established for EtCO_2_, with a view to predicting a decrease of over 10% in O_2_ER, and a increase over 10% in ScVO_2_.^22^ The threshold for statistical significance was set to *P* < 0.05. SPSS software (version 21, IBM, New York, NY) was used to perform statistical analysis.

## RESULTS

**FIGURE 1:**
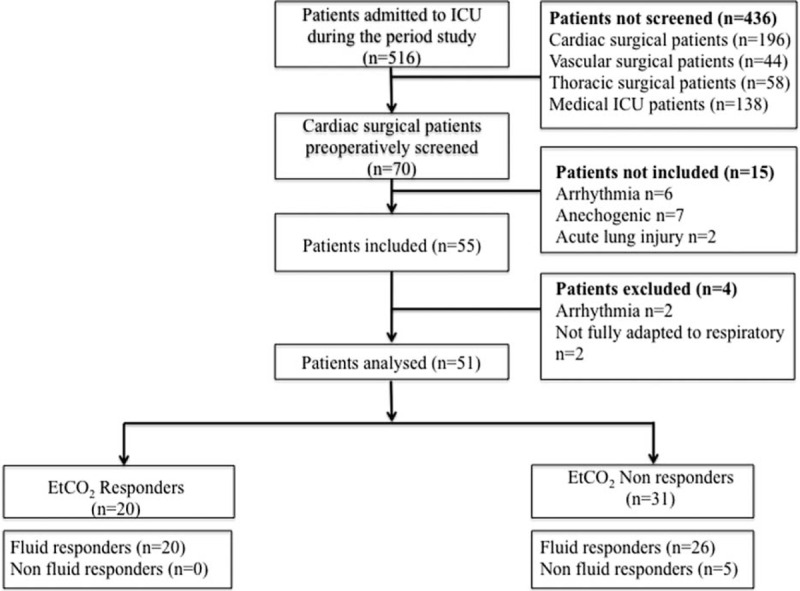
Study flow chart.

Fifty-one postoperative patients were analyzed after inclusion in the study (Figure 1, Table 1). The indications for VE were as follows: arterial hypotension (n = 34), oliguria (n = 4), and clinical signs of hypoperfusion (n = 13). Eighteen (35%) patients had hyperlactatemia. Indications for VE did not differ between EtCO_2_ responders and EtCO_2_ nonresponders (*P* > 0.05). Twenty (39%) of the 51 patients were classified as EtCO_2_ responders. All EtCO_2_ responders were also fluid responders and displayed a mean (95% CI) EtCO_2_ of 7% (6–9) during VE (Figure 1). Thirty-one patients were classified as EtCO_2_ nonresponders and displayed a mean (95% CI) change in EtCO_2_ of 0% (−1 to 1) during VE (Figure 1). Twenty-six of the EtCO_2_ nonresponders were fluid responders and 5 were fluid nonresponders. At baseline, prevalence of norepinephrine treatment did not differ between the 2 groups of patients (12 [39%] EtCO_2_ nonresponders vs 7 [35%] EtCO_2_ responders, *P* = 0.1). At baseline, SV variations with PLR did not differ between EtCO_2_ responders and EtCO_2_ nonresponders (*P* > 0.05, Table 2).

**TABLE 1:**
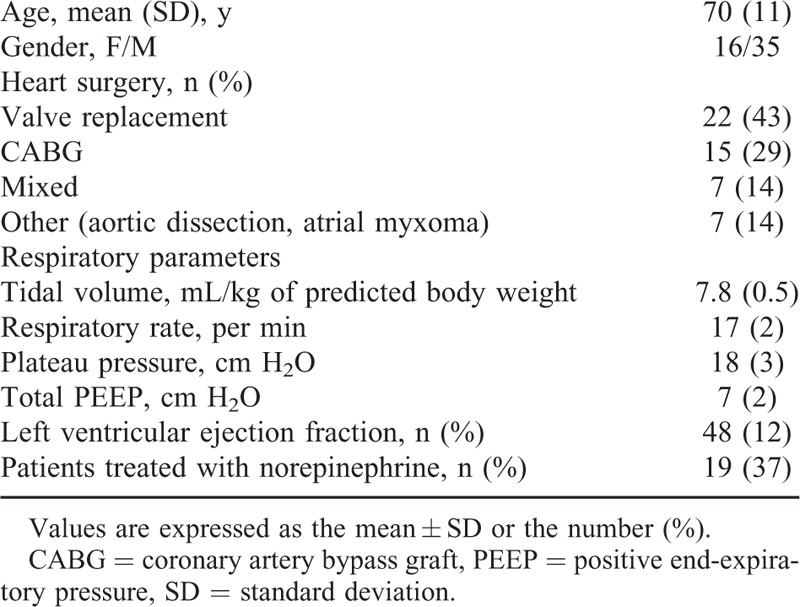
Characteristics of the Study Participants on Inclusion

**TABLE 2:**
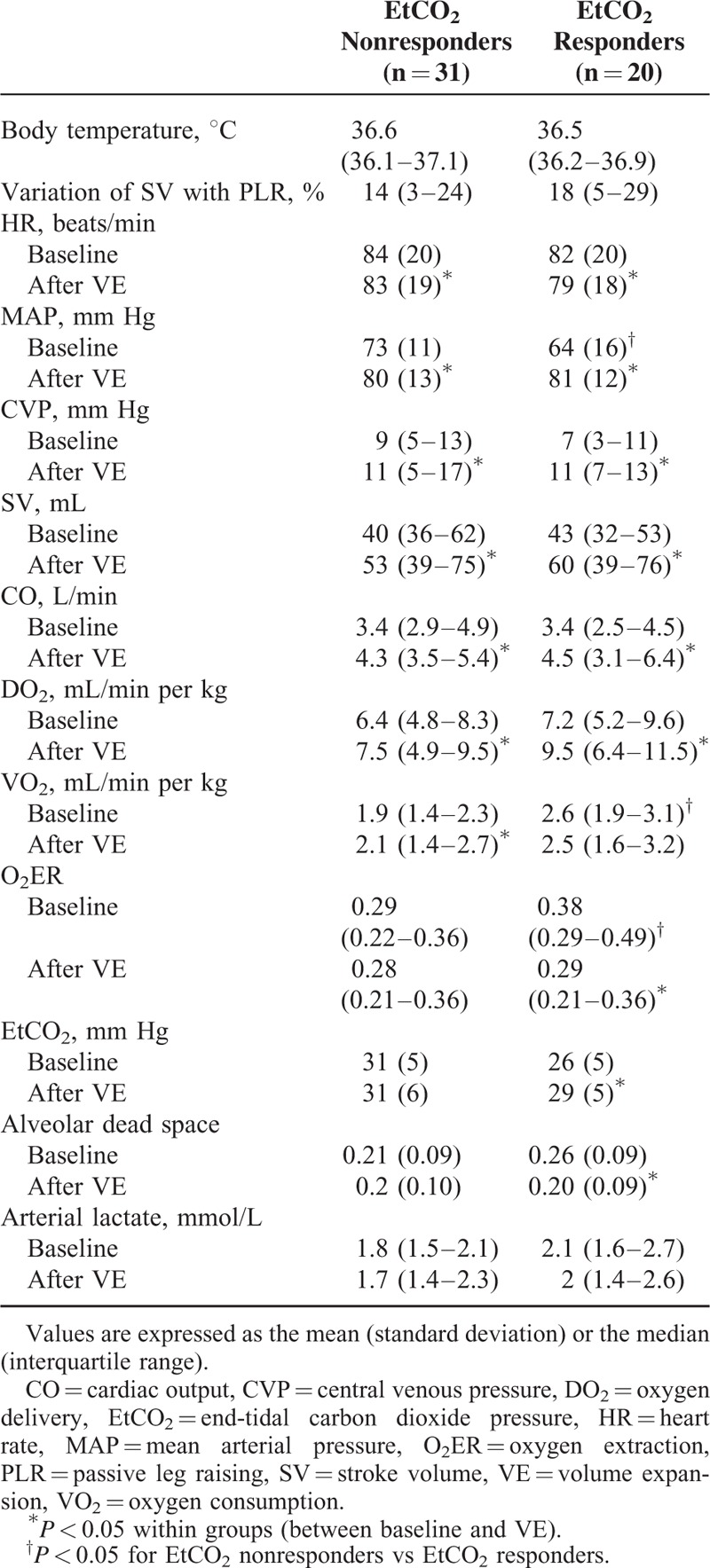
Comparison of Hemodynamic Parameters in Nonresponders, EtCO_2_ Nonresponders, and EtCO_2_ Responders

### Effect of VE on Hemodynamic and Blood Gas Parameters

In the study population as a whole, VE led to increases in MAP, CVP, SV, CO, EtCO_2_, DO_2_, PvO_2_, and ScvO_2_, and decreases in HR, PvCO_2_, O_2_ER, and alveolar dead space (Tables 2 and 3). At baseline, MAP, PvO_2_, and ScvO_2_ were lower and VO_2_ and O_2_ER were higher in EtCO_2_ responders than in EtCO_2_ nonresponders (regardless of the presence or absence of a fluid response in the latter; Tables 2 and 3).

**TABLE 3:**
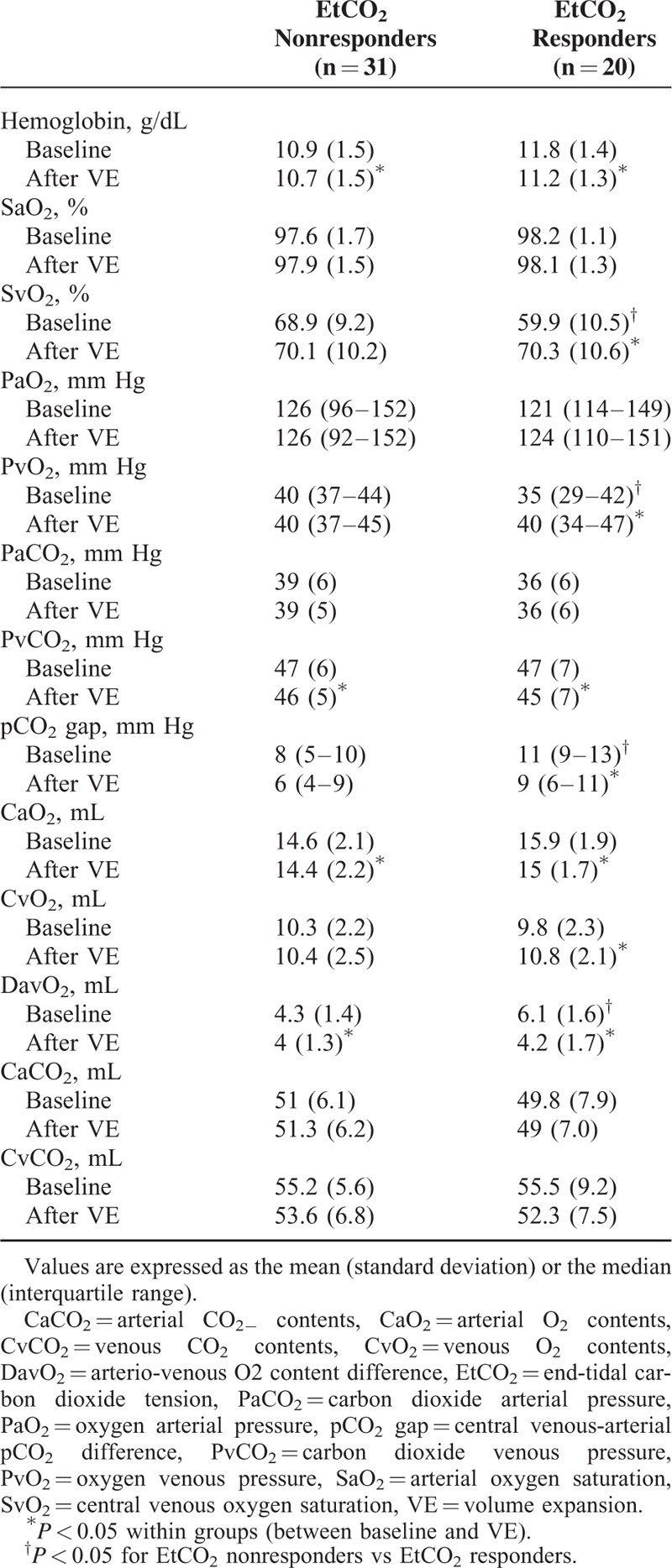
Comparison of Blood Gas Parameters in EtCO_2_ Nonresponders and EtCO_2_ Responder Groups

In EtCO_2_ nonresponders, VE led to increases in MAP, SV, CO, CVP, DO_2_, and VO_2_. PvCO_2_ decreased during VE. In EtCO_2_ responders, VE led to increases in MAP, SV, CO, CVP, EtCO_2_, PvO_2_, DO_2_, and ScvO_2_ and decreases in PvCO_2_, PcCO_2_ gap, O_2_ER, and alveolar dead space (Tables 2 and 3).

### Correlations Between Hemodynamic, Blood Gas Parameters, and EtCO_2_

In the overall population at baseline, EtCO_2_ was correlated with CO, DO_2_, and O_2_ER (*r* = 0.48, *P* = 0.001; *r* = 0.47, *P* = 0.001; and *r* = −0.42, *P* = 0.005, respectively). O_2_ER was not correlated with arterial lactate levels (*r* = 0.01, *P* = 0.99 but was correlated with PavCO_2_ (*r* = −0.46, *P* = 0.01). Changes in EtCO_2_ during VE were correlated with those in CO, PvO_2_, DO_2_, VO_2_, ScVO_2_, and O_2_ER (Table 4). A change of more than 4% in EtCO_2_ during VE predicted a decrease of more than 10% in the VO_2_/DO_2_ ratio with an area under the curve (95% CI) of 0.88 (0.77–0.96) (*P* < 0.0001), a sensitivity of 71% (53–85), a specificity of 94% (71–100), a positive likelihood ratio of 12, negative likelihood ratio of 0.31, a positive predictive value of 96, and a negative predictive value of 62. In the same way, change in EtCO_2_ during VE predicted an increase of more than 10% in ScVO_2_ with an area under the curve (95% CI) of 0.90 (0.78–0.97), *P* < 0.0001.

**TABLE 4:**
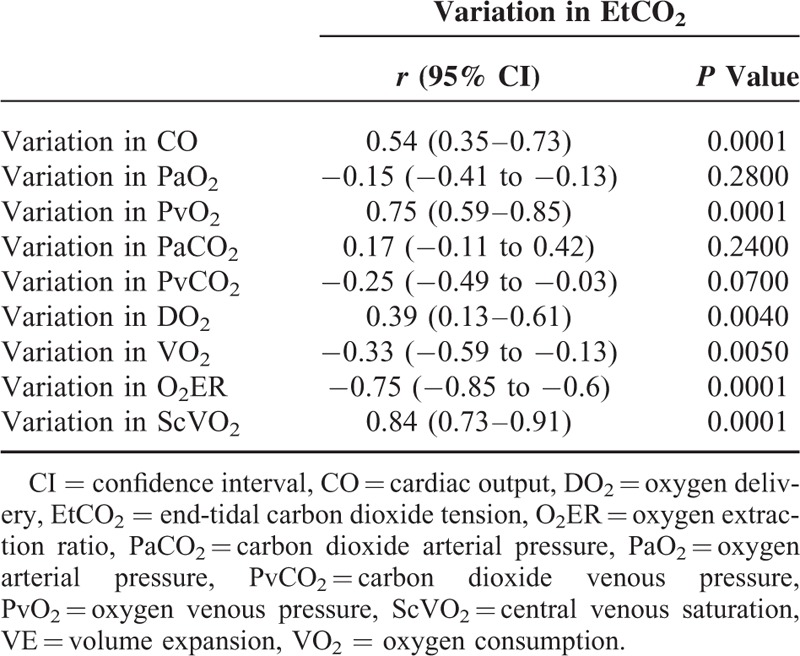
Correlations Between Variations in EtCO_2_, Hemodynamic Parameters, and Blood Gas Parameters During VE

## DISCUSSION

Our results demonstrated that during VE, the occurrence of concomitant increases in EtCO_2_ and CO depends on the relationship between VO_2_ and DO_2_; an increase in CO was not necessarily accompanied by an increase in EtCO_2_. Only patients with a high extraction ratio (i.e., low ScvO_2_ values) will display an increase in EtCO_2_. Thus, changes in EtCO_2_ during VE reflect changes in O_2_ER in response to a rise in DO_2_. Hence, during VE, EtCO_2_ may be a useful noninvasive indicator of changes in systemic oxygen supply dependency when fixed ventilation is maintained.

Several preclinical and clinical studies have demonstrated that EtCO_2_ can be used as a noninvasive, continuous measure of CO during low-flow states (cardiac arrest, hemorrhagic shock, cardiopulmonary resuscitation, circulatory shock, etc.).^9–11^ Similarly, EtCO_2_ has been shown to reflect changes in VO_2_ and VCO_2_ during hemorrhagic shock.^12,13^ Our present results demonstrated that EtCO_2_ may reflect changes in systemic oxygen supply associated with changes in CO during VE in nonseptic patients. All the patients in our study were postoperative sedated and nonhypothermic. Alveolar ventilation procedures and norepinephrine dosage did not change over the study period, and CO increased during VE. Even then, only 50% of fluid responders displayed an increase in EtCO_2_, thus EtCO_2_ was poorly correlated with changes in CO. Our results confirm previous findings in the operating theatre, where EtCO_2_ and CO were rather low.^14^ To determine the mechanisms by which increase in CO increase EtCO_2_ during VE, 1 would have to consider the study population and the effects of increase CO on blood gas parameters.

At baseline, EtCO_2_ responders had a lower MAP than EtCO_2_ nonresponders, whereas the 2 groups did not differ significantly in terms of preload status (i.e., variations in SV during PLR) and CO. Although EtCO_2_ responders and EtCO_2_ nonresponders did not differ in terms of DO_2_, the EtCO_2_ responders had a higher VO_2_ and thus a higher O_2_ER and lower ScVO_2_. VE led to an increase in CO (and thus DO_2_) and a decrease in PvCO_2_ in fluid responders. Nevertheless, VE in EtCO_2_ responders led to a recovery of DO_2_ in consistent with oxygen needs: the decrease in O_2_ER resulted in an increase in PvO_2_ and ScvO_2_. In contrast, EtCO_2_ nonresponsiveness was associated with an increase in DO_2_ and VO_2_ because baseline O_2_ER did not rise. Thus, concomitant increases in EtCO_2_ and CO may result from several different mechanisms.

Under steady-state conditions, alveolar CO_2_ elimination and therefore EtCO_2_ depend on several factors: CO_2_ production (VCO_2_, due to metabolism), alveolar ventilation (mechanical ventilation), pulmonary perfusion (CO), and V/Q matching. VCO_2_ depends on pulmonary elimination and metabolic production of CO_2_. The changes in EtCO_2_ in our population could not be explained by metabolic production of CO_2_ for several reasons. In a model of hemorrhagic shock, Dubin et al^13^ demonstrated that VCO_2_ could decrease EtCO_2_ during the period of VO_2_ supply dependency at low CO. The alterations in VCO_2_ were statistically significant for changes in CO, DO_2_, and VO_2_ values that were greater than those observed in our study. A further mechanism might be related to removal of peripheral tissue CO_2_ produced under anaerobic conditions.^23^ In the present study, the baseline O_2_ER values were below critical literature values at which tissue hypoxia was associated with anaerobic metabolism.^24^ Moreover, no inter and intra group difference was shown for CaCO_2_ and CvCO_2_. One can hypothesize that decrease (increases) in O_2_ER (ScVO_2_) and CO will decrease the venous blood's capacity to carry CO_2_ at a given PvO_2_, which in turn will offset the increase in CO_2_ delivery when CO rises.^25^

Thus, an increase in DO_2_ may decrease PvCO_2_ and increase CO_2_ delivery to the lung. At the same time, alveolar Vd/Vt fell in EtCO_2_ responders (despite constant minute ventilation) as a result of 2 mechanisms. The increases in PvO_2_ and CO may have improved alveolar perfusion pressure and the ventilation–perfusion ratio of the lung, which would tend to decrease PaCO_2_.^26,27^ In our population, changes in EtCO_2_ had good correlation with changes in PvO_2_ and ScVO_2_ whereas they were not associated to those in PaCO_2_ or PvCO_2_. These mechanisms may explain (at least in part) why EtCO_2_ did not change in EtCO_2_ nonresponders, whereas CO did.

In summary, EtCO_2_ responders had a low DO_2_ with regard to their VO_2_ resulting in higher O_2_ER (lower ScVO_2_). VE restored the relationship between VO_2_/DO_2_ through CO changes and increasing PvO_2_ and CO_2_ delivery to the lung, which improved the patients’ ventilation–perfusion ratio and thus increased EtCO_2_.

The present study had a number of limitations. The study population (patients after heart surgery) may have differed from septic shock patients. Most of our patients suffered from acute circulatory failure as a result of perioperative hypovolemia, whereas septic patients generally have acute circulatory failure that combines hypovolemia, changes in microvascular perfusion and cellular dysoxia. A patient's response to VE, the relationship between DO_2_ and VO_2_, and the extent of anaerobic metabolism may depend on the etiology of acute circulatory failure.^28–30^ We measured blood gas parameters from a central venous catheter and not from a pulmonary artery catheter. Although ScVO_2_ cannot give a precise absolute estimate of SvO_2_, it can serve as a guide to changes in SvO_2_ and VO_2_.^30,31^ Monnet et al used blood gas parameters from a central venous catheter to assess VO_2_, DO_2_, and their changes over time during fluid expansion in septic patients.^31^ In our study, changes in ScVO_2_ (measured) and O_2_ER (calculated) had good correlation (*r* = −0.89, *P* < 0.0001). Moreover, predictive values of EtCO_2_ changes during VE did not differ to predict an increase of ScVO_2_ or a decrease of VO_2_/DO_2_ ratio. Since we performed repeated measurements of blood gas levels, mathematical coupling cannot be ruled out. But De Backer et al demonstrated that during controlled conditions, VO_2_ calculated from hemodynamic data is a valid alternative to VO_2_ derived from respiratory gas measurements.^30,32^ The method used to calculate alveolar dead space was not standardized and may have introduced bias into the determination. The difference between EtCO_2_ and PaCO_2_ is altered in patients with altered ventilation/perfusion ratios (due to atelectasis, chronic heart failure, acute respiratory distress syndrome, etc.). In the present study, patients with chronic pulmonary disease or acute lung injury were excluded to limit this bias. Furthermore, these results cannot be extrapolated to EtCO_2_ changes that result from the administration of vasopressor drugs.^33^ EtCO_2_ changes seem small but they are similar to those used to predict fluid responsiveness.^34^ Lastly, given that we did not measure VCO_2_, we cannot rule out the occurrence of changes in metabolic production of CO_2_ during the VE-induced increase in CO.

## CONCLUSIONS

During VE, an increase in CO was not necessarily accompanied by an increase in EtCO_2_. Only patients with a high O_2_ER (i.e., low ScvO_2_ values) display an increase in EtCO_2_. Thus, EtCO_2_ changes during fluid challenge predict changes in O_2_ER (i.e., ScVO_2_) in response to an increase in DO_2_. EtCO_2_ may be a useful noninvasive indicator of changes in systemic oxygen supply dependency in operative patients when fixed ventilation is maintained.

## UNCITED REFERENCES

^18^.
